# Logarithmic-Domain Array Interpolation for Improved Direction of Arrival Estimation in Automotive Radars

**DOI:** 10.3390/s19102410

**Published:** 2019-05-27

**Authors:** Seongwook Lee, Seong-Cheol Kim

**Affiliations:** 1 Machine Learning Lab, AI & SW Research Center, Samsung Advanced Institute of Technology (SAIT), Gyeonggi-do 16678, Korea; 2 Department of Electrical and Computer Engineering and Institute of New Media & Communications (INMC), Seoul National University (SNU), Seoul 08826, Korea; sckim@maxwell.snu.ac.kr

**Keywords:** array interpolation, automotive radar, direction of arrival (DOA) estimation

## Abstract

In automotive radar systems, a limited number of antenna elements are used to estimate the angle of the target. Therefore, array interpolation techniques can be used for direction of arrival (DOA) estimation to achieve high angular resolution. In general, to generate interpolated array elements from original array elements, the method of linear least squares (LLS) is used. When the LLS method is used, the amplitudes of the interpolated array elements may not be equivalent to those of the original array elements. In addition, through the transformation matrix obtained from the LLS method, the phases of the interpolated array elements are not precisely generated. Therefore, we propose an array transformation matrix that generates accurate phases for interpolated array elements to improve DOA estimation performance, while maintaining constant amplitudes of the array elements. Moreover, to enhance the effect of our interpolation method, a power calibration method for interpolated received signals is also proposed. Through the simulation, we confirm that the array interpolation accuracy and DOA estimation performance of the proposed method are improved compared to those of the conventional method. Moreover, the performance and effectiveness of our proposed method are also verified using data obtained from the commercial radar system. Because the proposed method exhibits better performance when applied to actual measurement data, it can be utilized in commercial automotive radar systems.

## 1. Introduction

In recent years, radar systems have been installed in automobiles to detect targets located in multiple directions. Typically, automotive radar systems use frequencies in the 24-GHz or 77–81-GHz band. Since such a high frequency band is used, the miniaturization of the radar antenna system has become possible. In the automotive radar system, the number of receiving antenna elements is gradually decreasing to reduce the manufacturing cost of the radar. Therefore, various techniques have been proposed for accurately estimating the direction of arrival (DOA) of the target with the limited antenna elements as much as possible.

Array interpolation is one of the methods for improving the DOA estimation accuracy using a limited number of antenna elements. Several studies on enhanced array interpolation methods have been conducted [1,2,3,4,5]. In [1], the Taylor series approximation was used to generate interpolated array elements in a uniform circular array and achieved improved DOA estimation performance; however, because the order of the series is limited to one less than the maximum number of array elements, the approximation performance is not guaranteed for automotive radar systems that use only a few (e.g., four or eight) array elements. In addition, the norm-constrained least squares method was used to find the interpolated microphone array in [2], but the problem-solving process was heuristic because the proper norm constraint parameter was determined empirically. Recently, the effective interpolation method that changes the nonuniform co-prime array to the uniform linear array was proposed in [3]. Furthermore, the enhanced DOA estimation method using the characteristics of the covariance matrix generated by the virtual array interpolation for the co-prime array has been proposed in [4]. In [5], the authors improved the angle estimation performance by linearly predicting the received signals and virtually extending the array.

Among the various array interpolation methods, an array interpolation method that moves array elements from an original location to a desired location using a transformation matrix is widely used [6,7,8]. To this end, the linear least squares (LLS) method has been widely used to identify the proper transformation matrix [6,7,8,9,10,11,12]. However, a transformation matrix obtained by means of the LLS method is not the best solution for interpolating array elements. When this transformation matrix is applied, interpolated array elements are generated by linear combinations of original array elements. In this case, the amplitudes of interpolated array elements can be different from those of original array elements. If amplitude differences exist among the array elements, the performance of DOA estimation algorithms is degraded [13]. In addition, because the solution derived from the LLS method is obtained in the process of simultaneously minimizing differences in amplitudes and phases, the phase information of the interpolated array elements is not accurately formulated, which is a critical factor for DOA estimation.

Thus, in this paper, we propose a transformation matrix in the logarithmic domain for the array interpolation. We focus on minimizing the phase differences between the original and the interpolated array elements. First, we take logarithms for the array elements and extract the phase information from them. We then apply the LLS method to the logarithmic-domain matrices to find an appropriate transformation matrix. Finally, the interpolated array elements are generated by the new matrix, and the DOA estimation is conducted. Based on a comparison of interpolation errors of the proposed and conventional transformation methods, our array transformation method successfully interpolates newly-produced array elements with more elaborate phases. In addition, the proposed array transformation does not affect the amplitudes of the interpolated array elements; they are conserved even after the transformation. Moreover, for a given antenna array, the proposed transformation matrix for that array is calculated and stored (offline) in advance. Thus, we do not have to calculate the transformation matrix in real time.

We also extend the proposed array interpolation scheme to received signal interpolation. When we use the transformation matrices obtained by the LLS method and our proposed method, the powers of the interpolated received signals are not uniform over all array elements. In this case, the effect of the array interpolation and the performance of the DOA estimation are not fully ensured. Thus, to mitigate this problem, we also propose a calibration method for the interpolated received signal powers. The simulation results confirm that our proposed method performs DOA estimation better than the conventional array interpolation method. In addition, based on actual measurement data acquired using an automotive radar, our method shows improved angular resolution and estimation performance. The proposed method can be effectively used in a radar system using a small number of array elements.

The remainder of this paper is organized as follows. In Section 2, we introduce the basic signal model for the array antenna, as well as the conventional array interpolation technique using the LLS method. Next, the proposed array interpolation method is described in Section 3. In this section, we also propose a method of calibrating the interpolated received signals for more accurate DOA estimation. Simulation and measurement results are provided in Section 4 and Section 5, respectively. We conclude this paper in Section 6.

## 2. Fundamentals of Array Interpolation

### 2.1. Signal Model for the Array Antenna

We assume that signals, coming from *K* directions of $\theta_{1},\;\theta_{2},\;\cdots ,\;\theta_{K}$, are incident on *N* linearly placed array elements. The location of the array elements is $\left[d_{1},\;d_{2},\;\cdots ,\;d_{N}\right]$ and $\theta_{k}\;\left(k=1,\;2,\;\cdots ,\;K\right)$ is defined from the boresight direction of the array antenna. Assuming far-field narrow-band signal sources, the received signal vector of the array at time *t* can be expressed as:

(1) $$\begin{array}{ccc} X(t) & = & A\times S(t)+N(t)\\ & = & {\left[x_{1}\left(t\right),\;x_{2}\left(t\right),\;\cdots ,\;x_{N}\left(t\right)\right]}^{T} \end{array}$$

where ${\left[\cdot \right]}^{T}$ denotes the vector transpose operator and $A=[a\left(\theta_{1}\right),\;a\left(\theta_{2}\right),\;\cdots ,\;a\left(\theta_{K}\right)]$ is the steering matrix composed of the steering vectors $a(\theta_{k})$ given by:

(2) $$\begin{array}{c} a\left(\theta_{k}\right)={\left[e^{j\frac{2\pi }{\lambda }d_{1}\sin\theta_{k}},\;e^{j\frac{2\pi }{\lambda }d_{2}\sin\theta_{k}},\;\cdots ,\;e^{j\frac{2\pi }{\lambda }d_{N}\sin\theta_{k}}\right]}^{T}. \end{array}$$

λ denotes the wavelength corresponding to the carrier frequency. $S\left(t\right)={\left[s_{1}\left(t\right),\;s_{2}\left(t\right),\;\cdots ,\;s_{K}\left(t\right)\right]}^{T}$ is the incident signal vector, where $s_{k}\left(t\right)\;\left(k=1,\;2,\;\cdots ,\;K\right)$ is the complex amplitude of the incident signal from the *k*^th^ signal source at time *t*. These amplitudes are assumed to be zero-mean complex Gaussians and uncorrelated with each other. In addition, the noise vector $N\left(t\right)={\left[n_{1}\left(t\right),\;n_{2}\left(t\right),\;\cdots ,\;n_{N}\left(t\right)\right]}^{T}$ is assumed to be the zero-mean complex Gaussian vector, and its components are also uncorrelated with each other. The samples from S(t) and N(t) are also assumed to be uncorrelated with each other. If there is a correlation between the signals received from the radar system, the correlation can be lowered through signal processing techniques such as spatial smoothing [14].

### 2.2. Conventional Array Interpolation Method

In this section, we briefly introduce the conventional array interpolation technique using the transformation matrix derived from the LLS method. For the field of view (FOV) of an automotive radar, which is expressed as:

(3) $$\begin{array}{c} \Theta =\{\theta_{p}|\theta_{p}=\theta_{L}+\left(p-1\right)\times \frac{\theta_{R}-\theta_{L}}{P-1},\;p=1,\;2,\;\cdots ,\;P\}, \end{array}$$

we find a suitable matrix that transforms the original array elements into the interpolated array elements ($\theta_{L}$ and $\theta_{R}$ are angles that indicate the left and the right boundaries of the FOV). In this range, the steering matrix of the original array elements can be given as:

(4) $$\begin{array}{c} A\left(\Theta \right)=[a\left(\theta_{L}\right),\;a\left(\theta_{L}+\Delta \theta \right),\;\cdots ,\;a\left(\theta_{L}+\left(P-2\right)\Delta \theta \right),\;a\left(\theta_{R}\right)] \end{array}$$

where $\Delta \theta =\frac{\theta_{R}-\theta_{L}}{P-1}$ is the angle step size. Then, if we want to interpolate array elements in the location $\left[g_{1},\;g_{2},\;\cdots ,\;g_{M}\right]$, the steering matrix of the interpolated array elements is determined as:

(5) $$\begin{array}{c} B\left(\Theta \right)=[b\left(\theta_{L}\right),\;b\left(\theta_{L}+\Delta \theta \right),\;\cdots ,\;b\left(\theta_{L}+\left(P-2\right)\Delta \theta \right),\;b\left(\theta_{R}\right)] \end{array}$$

where $b\left(\theta_{p}\right)={\left[e^{j\frac{2\pi }{\lambda }g_{1}\sin\theta_{p}},\;e^{j\frac{2\pi }{\lambda }g_{2}\sin\theta_{p}},\;\cdots ,\;e^{j\frac{2\pi }{\lambda }g_{M}\sin\theta_{p}}\right]}^{T}$ (p=1,2,⋯,P). Assuming that a matrix T transforms the original steering matrix to the interpolated steering matrix, it can be expressed as:

(6) $$\begin{array}{c} B(\Theta )=T\times A(\Theta ). \end{array}$$

To find the proper transformation matrix T, the least squares method is used as:

(7) $$\begin{array}{c} T^{*}=\arg\min_{T}\left({∥B(\Theta )-T\times A(\Theta )∥}_{F}\right) \end{array}$$

where ${∥\cdot ∥}_{F}$ denotes the Frobenius matrix norm. Then, based on the method of LLS, the transformation matrix can be determined as:

(8) $$\begin{array}{c} T^{*}=B\left(\Theta \right)A{\left(\Theta \right)}^{H}{\left(A\left(\Theta \right)A{\left(\Theta \right)}^{H}\right)}^{-1} \end{array}$$

where ${\left[\cdot \right]}^{H}$ denotes the Hermitian matrix operator. Finally, from (6) and (8), the estimate of B(Θ) is given as:

(9) $$\begin{array}{cc} {\hat{B}}^{T^{*}}\left(\Theta \right) & =T^{*}\times A\left(\Theta \right)\\ & =B\left(\Theta \right)A{\left(\Theta \right)}^{H}{\left(A\left(\Theta \right)A{\left(\Theta \right)}^{H}\right)}^{-1}\times A\left(\Theta \right). \end{array}$$

In addition, the ${\left(m,\;p\right)}^{\mathrm{th}}$ element of the matrix ${\hat{B}}^{T^{*}}\left(\Theta \right)$ can be expressed as:

(10) $$\begin{array}{ccc} {\hat{B}}_{\left(m,\;p\right)}^{T^{*}}\left(\Theta \right) & = & \sum_{n=1}^{N}\left\{{T^{*}}_{\left(m,\;n\right)}\times A_{\left(n,\;p\right)}\left(\Theta \right)\right\}\\ & = & \sum_{n=1}^{N}\left\{{T^{*}}_{\left(m,\;n\right)}\times e^{j\frac{2\pi }{\lambda }d_{n}\sin\theta_{p}}\right\},\\ & & \forall m\in \{1,\;2,\;\cdots ,\;M\},\;\forall p\in \{1,\;2,\;\cdots ,\;P\} \end{array}$$

where ${T^{*}}_{\left(m,\;n\right)}$ and $A_{\left(n,\;p\right)}\left(\Theta \right)$ denote the ${\left(m,\;n\right)}^{\mathrm{th}}$ and the ${\left(n,\;p\right)}^{\mathrm{th}}$ elements of the matrices $T^{*}$ and A(Θ), respectively.

Through this transformation matrix, received signals for the interpolated array elements can also be generated, which are defined as:

(11) $$\begin{array}{ccc} \hat{Y}\left(t\right) & = & T^{*}\times X\left(t\right)\\ & = & [{\hat{y}}_{1}\left(t\right),\;{\hat{y}}_{2}\left(t\right),\;\cdots ,\;{\hat{y}}_{M}\left(t\right)]. \end{array}$$

By utilizing these interpolated received signals, the authors of [6,7,8,9,10,11,12] conducted improved DOA estimations.

## 3. Logarithmic-Domain Array Interpolation

### 3.1. Proposed Array Interpolation Method

When we use the conventional transformation matrix for array interpolation, a major problem occurs. Based on the transformation matrix obtained from the LLS method, interpolated array elements are generated by linear combinations of original array elements. In this case, the amplitudes of the interpolated array elements may not be equivalent to those of the original array elements. In other words, $\left|{\hat{B}}_{\left(m,\;p\right)}^{T^{*}}\left(\Theta \right)\right|$ does not always become unity. When the amplitudes of each array element are not uniform over the entire array, DOA estimation performance is degraded [13]. In addition, based on the solution derived from the LLS method, the phases of the interpolated array elements are not precisely generated. For DOA estimation, the phase information of the interpolated array elements is critical. Therefore, in this section, we propose a more effective array interpolation method that minimizes the phase differences between the original and the interpolated array elements while maintaining the equivalent amplitudes of the array elements.

For the elements of the steering matrices on both sides of (6), we take logarithms such as:

(12) $$\begin{array}{ccc} LOG(A(\Theta )) & = & \left(\begin{array}{ccc} \log(A_{\left(1,\;1\right)}\left(\Theta \right)) & \cdots & \log(A_{\left(1,\;P\right)}\left(\Theta \right))\\ \log(A_{\left(2,\;1\right)}\left(\Theta \right)) & \cdots & \log(A_{\left(2,\;P\right)}\left(\Theta \right))\\ \vdots & \ddots & \vdots \\ \log(A_{\left(N,\;1\right)}\left(\Theta \right)) & \cdots & \log(A_{\left(N,\;P\right)}\left(\Theta \right)) \end{array}\right),\\ LOG(B(\Theta )) & = & \left(\begin{array}{ccc} \log(B_{\left(1,\;1\right)}\left(\Theta \right)) & \cdots & \log(B_{\left(1,\;P\right)}\left(\Theta \right))\\ \log(B_{\left(2,\;1\right)}\left(\Theta \right)) & \cdots & \log(B_{\left(2,\;P\right)}\left(\Theta \right))\\ \vdots & \ddots & \vdots \\ \log(B_{\left(M,\;1\right)}\left(\Theta \right)) & \cdots & \log(B_{\left(M,\;P\right)}\left(\Theta \right)) \end{array}\right), \end{array}$$

where LOG(·) denotes the operator that takes logarithms for each element in the matrix and $B_{\left(m,\;p\right)}\left(\Theta \right)$ indicates the ${\left(m,\;p\right)}^{\mathrm{th}}$ element of the matrix B(Θ). All elements in matrices LOG(A(Θ)) and LOG(B(Θ)) have pure imaginary values. Then, in the logarithmic domain, we find a proper transformation matrix V that transforms LOG(A(Θ)) to LOG(B(Θ)), which is expressed as:

(13) $$\begin{array}{c} LOG(B(\Theta ))=V\times LOG(A(\Theta )). \end{array}$$

As in the original domain, the appropriate matrix V can be found using the LLS method, and the solution is given as:

(14) $$\begin{array}{c} V^{*}=LOG\left(B\left(\Theta \right)\right){LOG(A(\Theta ))}^{H}\times \;{\left\{LOG\left(A\left(\Theta \right)\right){LOG(A(\Theta ))}^{H}\right\}}^{-1}. \end{array}$$

This matrix $V^{*}$ effectively transforms the phases of the original array elements into those of the interpolated array elements. However, since the matrix is defined in the logarithmic domain, it cannot be directly applied to the original array elements as in (9). In other words, this transformation matrix cannot be expressed with a linear operator. Instead, it can be written with the original array elements as:

(15) $$\begin{array}{c} {\hat{B}}_{\left(m,\;p\right)}^{V^{*}}\left(\Theta \right)=\prod_{n=1}^{N}{\left\{A_{\left(n,\;p\right)}\left(\Theta \right)\right\}}^{{V^{*}}_{\left(m,\;n\right)}},\\ \forall m\in \left\{1,\;2,\;\cdots ,\;M\right\},\;\forall p\in \left\{1,\;2,\;\cdots ,\;P\right\} \end{array}$$

where ${\hat{B}}_{\left(m,\;p\right)}^{V^{*}}\left(\Theta \right)$ is the ${\left(m,\;p\right)}^{\mathrm{th}}$ element of the newly-interpolated steering matrix ${\hat{B}}^{V^{*}}\left(\Theta \right)$ and ${V^{*}}_{\left(m,\;n\right)}$ indicates the ${\left(m,\;n\right)}^{\mathrm{th}}$ element of the matrix $V^{*}$.

The conventional transformation matrix in (8) formulates the interpolated array elements with linear combinations of the original array elements. However, this new transformation matrix generates only the phase information of the interpolated array elements using combinations of the phases of the original array elements. In other words, based on our transformation, it conserves the amplitudes of the original array elements in the interpolated array elements because:

(16) $$\begin{array}{ccc} \left|{\hat{B}}_{\left(m,\;p\right)}^{V^{*}}\left(\Theta \right)\right| & = & \left|\prod_{n=1}^{N}{\left\{A_{\left(n,\;p\right)}\left(\Theta \right)\right\}}^{{V^{*}}_{\left(m,\;n\right)}}\right|\\ & = & \left|e^{\sum_{n=1}^{N}\left\{j\frac{2\pi }{\lambda }d_{n}\sin\theta_{p}{V^{*}}_{\left(m,\;n\right)}\right\}}\right|=1,\\ & & \forall m\in \left\{1,\;2,\;\cdots ,\;M\right\},\;\forall p\in \left\{1,\;2,\;\cdots ,\;P\right\}. \end{array}$$

Therefore, the proposed array transformation affects only the phases of the interpolated array elements and generates more accurate phases for the interpolated array elements. The transformation matrix $T^{*}$ does not preserve the amplitudes of the original array elements because $\left|{\hat{B}}_{\left(m,\;p\right)}^{T^{*}}\left(\Theta \right)\right|$ is not always unity. Thus, the interpolation accuracy derived from the new transform matrix $V^{*}$ is higher than that from the conventional matrix $T^{*}$.

### 3.2. Enhanced Received Signal Interpolation

Similar to the received signal interpolation in (11), received signals of the interpolated array elements with the transformation matrix $V^{*}$ are expressed as:

(17) $$\begin{array}{c} {\hat{z}}_{m}\left(t\right)=\prod_{n=1}^{N}{\left\{x_{n}\left(t\right)\right\}}^{{V^{*}}_{\left(m,\;n\right)}}\in \hat{Z}\left(t\right),\\ \forall m\in \{1,\;2,\;\cdots ,\;M\}. \end{array}$$

Using $\hat{Z}\left(t\right)=\left[{\hat{z}}_{1}\left(t\right),\;{\hat{z}}_{2}\left(t\right),\;\cdots ,\;{\hat{z}}_{M}\left(t\right)\right]$, we conduct the DOA estimation and can achieve improved performance compared to the estimation using $\hat{Y}\left(t\right)$.

For a much better DOA estimation, we also consider the power of the received signals. When we use the interpolated received signal vectors, $\hat{Y}\left(t\right)$ and $\hat{Z}\left(t\right)$, power differences exist among the interpolated received signals. In other words,

(18) $$\begin{array}{c} {\left|{\hat{y}}_{m}\left(t\right)\right|}^{2}={\left|{\hat{y}}_{m^{\prime }}\left(t\right)\right|}^{2}\;\mathrm{and}\;{\left|{\hat{z}}_{m}\left(t\right)\right|}^{2}={\left|{\hat{z}}_{m^{\prime }}\left(t\right)\right|}^{2}\\ \left(\mathrm{for}\;m\neq m^{\prime },\;m^{\prime }\in \left\{1,\;2,\;\cdots ,\;M\right\}\right) \end{array}$$

does not always hold, because:

(19) $$\begin{array}{c} \sum_{n=1}^{N}{\left|{T^{*}}_{\left(m,\;n\right)}\right|}^{2}\neq \sum_{n=1}^{N}{\left|{T^{*}}_{\left(m^{\prime },\;n\right)}\right|}^{2},\;\;\;\;\;\\ {T^{*}}_{\left(m,\;n\right)}\bar{{T^{*}}_{\left(m,\;n^{\prime }\right)}}\neq {T^{*}}_{\left(m^{\prime },\;n\right)}\bar{{T^{*}}_{\left(m^{\prime },\;n^{\prime }\right)}}\;\mathrm{and}\;\;\\ \sum_{n=1}^{N}{V^{*}}_{\left(m,\;n\right)}\neq \sum_{n=1}^{N}{V^{*}}_{\left(m^{\prime },\;n\right)}\;\;\;\;\;\;\;\;\\ (\mathrm{for}\;n\neq n^{\prime },\;n^{\prime }\in \left\{1,\;2,\;\cdots ,\;N\right\},\;\\ \;\mathrm{for}\;m\neq m^{\prime },\;m^{\prime }\in \left\{1,\;2,\;\cdots ,\;M\right\})\; \end{array}$$

(see Appendix A) where $\bar{\;\cdot \;}$ denotes the complex conjugate of a complex number. This power imbalance can cause performance degradation in the DOA estimation [13]. In our proposed method, the amplitudes of ${\hat{B}}_{\left(m,\;p\right)}^{V^{*}}\left(\Theta \right)$ are equivalent for all array elements. However, it is not directly related to the powers of the interpolated received signals, and power differences exist among the interpolated received signals. Therefore, to mitigate this problem, we propose an effective compensation method to formulate the received signals of each interpolated array element such that they have similar power levels while maintaining the effect of our proposed phase interpolation method. In other words, the compensated received signal is given as:

(20) $$\begin{array}{c} {\hat{w}}_{m}\left(t\right)={\left\{\prod_{n\in N_{\left(m\right)}}\left|x_{n}\left(t\right)\right|\right\}}^{\frac{1}{\left|N_{\left(m\right)}\right|}}\times \;\exp\left(j\sum_{n\in N_{\left(m\right)}}\left\{{V^{*}}_{\left(m,\;n\right)}∠x_{n}\left(t\right)\right\}\right)\in \hat{W}\left(t\right) \end{array}$$

where:

(21) $$\begin{array}{c} N_{\left(m\right)}=\left\{n^{*}|n^{*}=\arg_{n}\left({V^{*}}_{\left(m,\;n\right)}\neq 0\right),\;n=1,\;2,\;\cdots ,\;N\right\},\\ \forall m\in \left\{1,\;2,\;\cdots ,\;M\right\}, \end{array}$$

and $\left|N_{\left(m\right)}\right|$ denotes the cardinality of the set $N_{\left(m\right)}$. When comparing ${\hat{w}}_{m}\left(t\right)$ with ${\hat{z}}_{m}\left(t\right)$, the interpolated phase of ${\hat{w}}_{m}\left(t\right)$ is the same as that of ${\hat{z}}_{m}\left(t\right)$. Therefore, the phase interpolation effect from the transformation matrix $V^{*}$ is maintained. In addition, when we use this compensated interpolated received signal, the following equation is always established as:

(22) $$\begin{array}{ccc} {\left|{\hat{w}}_{m}\left(t\right)\right|}^{2} & = & {\left|{\hat{w}}_{m^{\prime }}\left(t\right)\right|}^{2}\;\\ (\mathrm{for}\;m & \neq & m^{\prime },\;m^{\prime }\in \left\{1,\;2,\;\cdots ,\;M\right\}), \end{array}$$

because:

(23) $$\begin{array}{ccc} {\left|{\hat{w}}_{m}\left(t\right)\right|}^{2} & = & {\left\{\prod_{n\in N_{\left(m\right)}}\left|x_{n}\left(t\right)\right|\right\}}^{\frac{2}{\left|N_{\left(m\right)}\right|}}\\ & = & {\left\{\underset{\left|N_{\left(m\right)}\right|}{\underset{︸}{\left|x_{1}\left(t\right)\right|\times \cdots \times \left|x_{N}\left(t\right)\right|}}\right\}}^{\frac{2}{\left|N_{\left(m\right)}\right|}}\\ & = & {\left\{\underset{\left|N_{\left(m\right)}\right|}{\underset{︸}{{\left|x_{1}\left(t\right)\right|}^{2}\times \cdots \times {\left|x_{N}\left(t\right)\right|}^{2}}}\right\}}^{\frac{1}{\left|N_{\left(m\right)}\right|}}\\ & ≅ & \gamma ,\;\forall m\in \left\{1,\;2,\;\cdots ,\;M\right\}. \end{array}$$

In other words, the powers of the interpolated received signals are nearly equivalent among the array elements. Thus, if we use the received signal vector $\hat{W}\left(t\right)$ for the DOA estimation, we can achieve more enhanced performance than when using $\hat{Y}\left(t\right)$ and $\hat{Z}\left(t\right)$.

## 4. Simulation Results

Many studies have been conducted on the location in which to interpolate array elements to improve the accuracy of DOA estimation algorithms. In [6,8], the authors located the interpolated array elements that minimized interpolation errors within given conditions. In addition, the array searching method proposed in [12] revealed enhanced DOA estimation accuracy with the interpolated array. However, this method was deemed too heuristic and time consuming. In this paper, to verify the DOA estimation accuracy resulting from our proposed interpolation method, we transformed the original array elements to the minimum-redundancy linear arrays, while maintaining identical apertures. In general, minimum-redundancy linear arrays show the maximum resolution for a given number of array elements by minimizing the number of redundant spacings in the array [15,16]. Moreover, previous studies have reported that non-uniform linear arrays perform better at DOA estimation than do uniform linear arrays that have the same apertures [17,18]. Therefore, in our simulation, by transforming the original array to the non-uniform minimum-redundancy linear array, we analyzed the performance improvement in the DOA estimation.

In the simulation, we used four array elements (N=4) that are widely used in automotive long-range radar (LRR). The location of the original array elements was $\left[d_{1},\;d_{2},\;d_{3},\;d_{4}\right]=\left[0,\;2\lambda ,\;4\lambda ,\;6\lambda \right]$. It is well known that the minimum-redundancy linear array location of four array elements is [0,1,4,6] [15,16]. Thus, using the array transformation matrices, we interpolated array elements in the location $\left[g_{1},\;g_{2},\;g_{3},\;g_{4}\right]=\left[0,\;1\lambda ,\;4\lambda ,\;6\lambda \right]$. Here, we assumed that two targets were located at $\left[\theta_{1},\;\theta_{2}\right]=\left[-3.5^{\circ },\;2.5^{\circ }\right]$ and adopted the Bartlett method [14] as the DOA estimation algorithm. In addition, the signal-to-noise ratio (SNR) at the array elements was set to 10 dB, and 1000 time samples were used to construct the correlation matrix used in the Bartlett algorithm. The FOV was given as $\Theta =\{\theta_{p}|\theta_{p}=-10^{\circ }+\left(p-1\right)\times 0.1^{\circ },\;p=1,\;2,\;\cdots ,\;201\}$, which was equivalent to the FOV of the LRR. Since $T^{*}$ and $V^{*}$ were calculated and stored only once when the number of array elements and the FOV were given, the stored values can be used repeatedly without having to identify another $T^{*}$ and $V^{*}$.

First, under these simulation conditions, we calculated two types of interpolation errors, which were given as:

(24) $$\begin{array}{ccc} E^{U} & = & {∥B\left(\Theta \right)-{\hat{B}}^{U}\left(\Theta \right)∥}_{F}^{2},\\ E_{phase}^{U} & = & {∥∠B\left(\Theta \right)-∠{\hat{B}}^{U}\left(\Theta \right)∥}_{F}^{2},\\ & & \;\forall U\in \{T^{*},\;V^{*}\}. \end{array}$$

**Figure 1:**
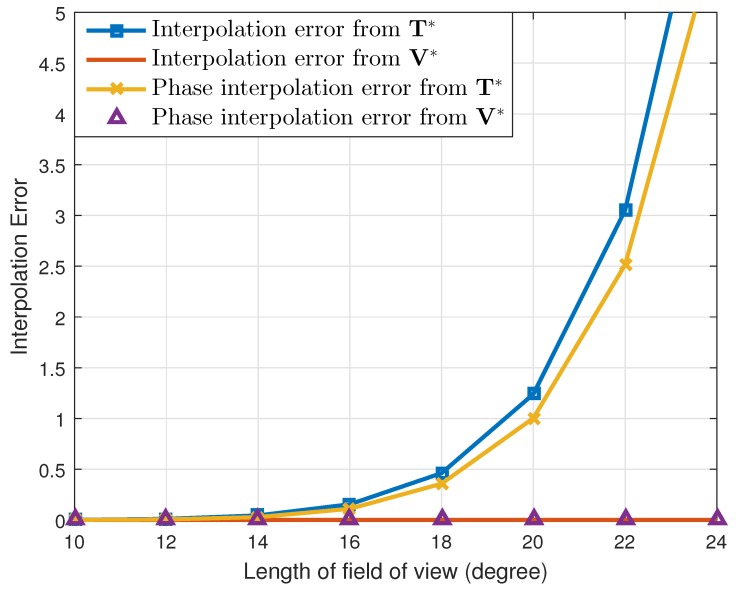
Two types of interpolation errors from $T^{*}$ and $V^{*}$.

The smaller the error values were calculated based on (24), the more accurate the array interpolation was conducted. For both transformation matrices, $T^{*}$ and $V^{*}$, we calculated the interpolation errors by changing the size of the FOV. The result is given in Figure 1. As the figure shows, the interpolation errors calculated from $V^{*}$ were almost close to zero. In addition, for the FOV of the LRR (i.e., the size of the FOV being $20^{\circ }$), the errors are given as $\left[E^{T^{*}},\;E^{V^{*}}\right]=\left[1.240,\;4.719\times 10^{-28}\right]$ and $\left[E_{phase}^{T^{*}},\;E_{phase}^{V^{*}}\right]=\left[1.004,\;4.700\times 10^{-28}\right]$. Therefore, judging from both types of interpolation errors, our proposed array transformation matrix ${\hat{B}}^{V^{*}}\left(\Theta \right)$ was more approximate to B(Θ) than was ${\hat{B}}^{T^{*}}\left(\Theta \right)$. In other words, the interpolated array elements were accurately generated when the proposed interpolation method was employed. For larger FOV sizes, the interpolation errors of the conventional method became larger because the interpolation matrix was calculated more accurately when the DOA range of the targets was tightly within the FOV.

**Figure 2:**
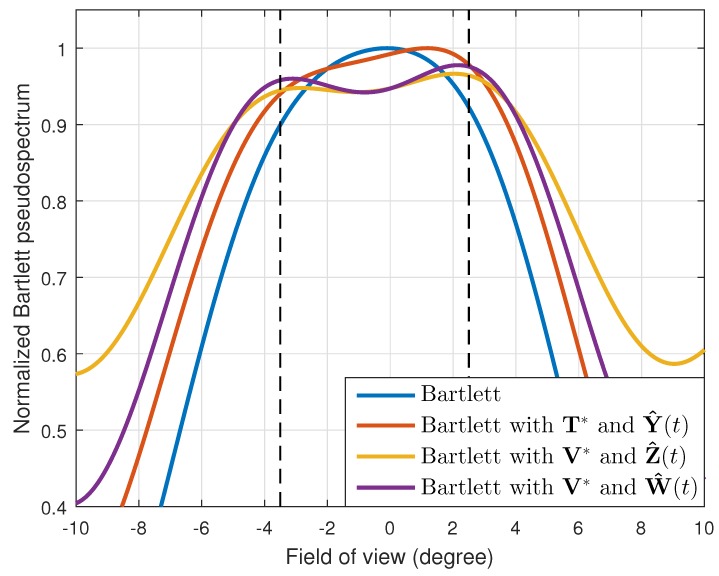
Normalized Bartlett pseudospectrums for two adjacent targets located at $\left[-3.5^{\circ },\;2.5^{\circ }\right]$.

Using these transformation matrices, we formulated the received signals and conducted the DOA estimation. As shown in Figure 2, with the original received signals, the Bartlett method could not resolve the two targets, and the estimated DOA was $-0.1^{\circ }$. In general, when we used four array elements with 2λ spacing, the half-power beamwidth became $6.5^{\circ }$. Therefore, the difficulty in distinguishing those given DOAs was reasonable. Even with the interpolated received signals from $T^{*}$, two different DOAs were not estimated, and the estimated DOA was $1.2^{\circ }$, which was not the exact value. However, with the interpolated received signals from $V^{*}$, the Bartlett method showed enhanced angular resolution, and we can find two different DOAs such as $\left[-2.8^{\circ },\;2.0^{\circ }\right]$. Moreover, when using the interpolated received signal vector with the power calibration, $\hat{W}\left(t\right)$, the best estimation result was achieved, and the estimated DOA values were $\left[-3.1^{\circ },\;2.2^{\circ }\right]$, which were close to the actual DOA values.

**Figure 3:**
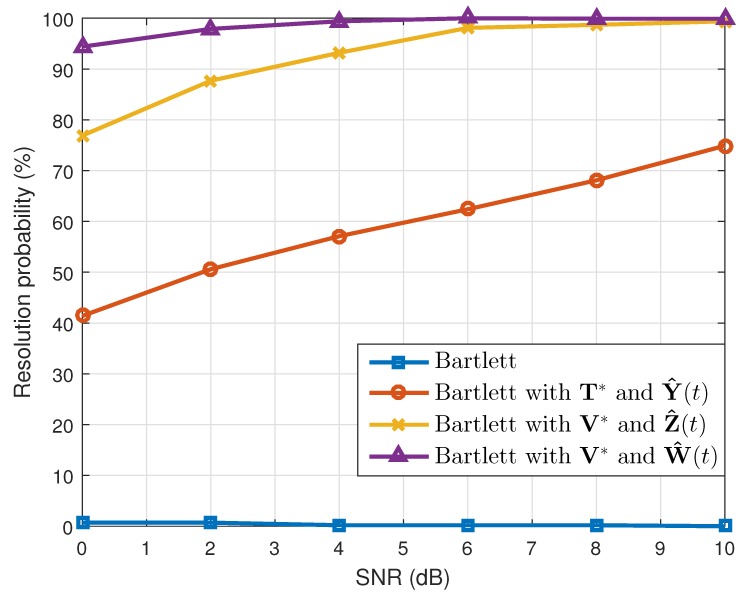
Resolution probabilities versus SNR (N=4).

**Figure 4:**
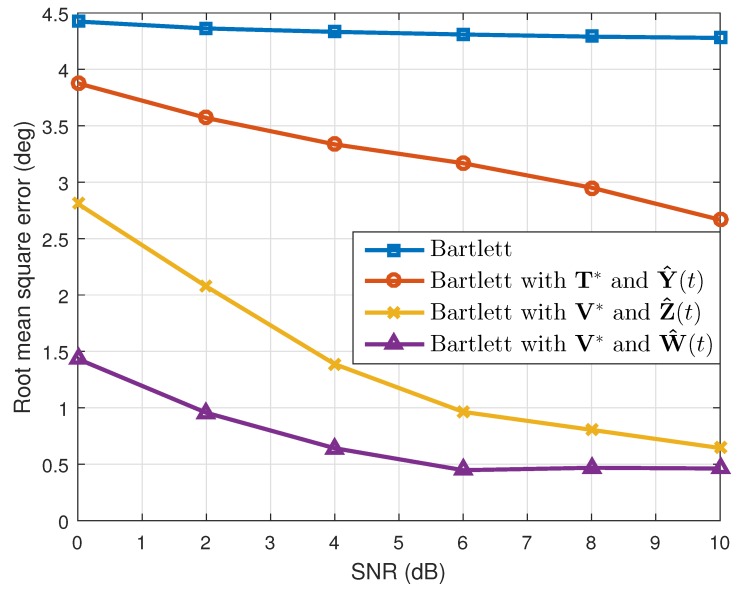
Root mean squared errors versus SNR (N=4).

**Figure 5:**
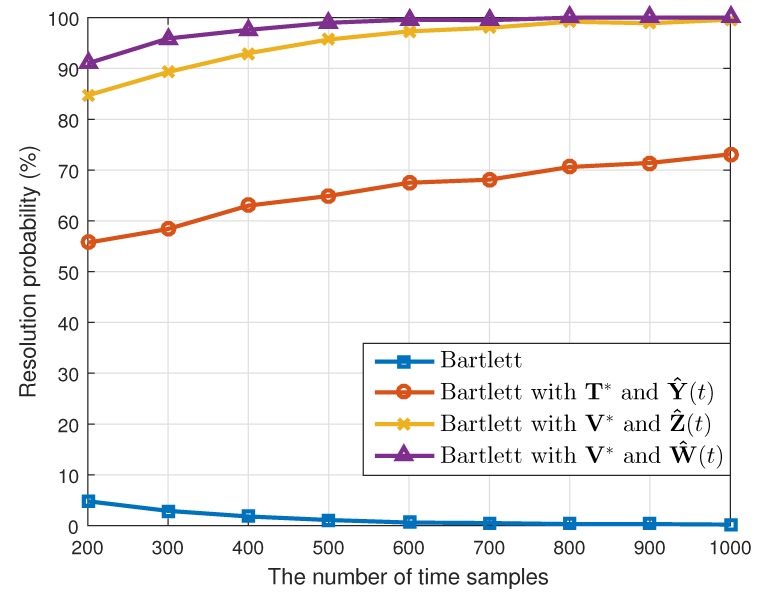
Resolution probabilities versus the number of time samples (SNR is 10 dB).

**Figure 6:**
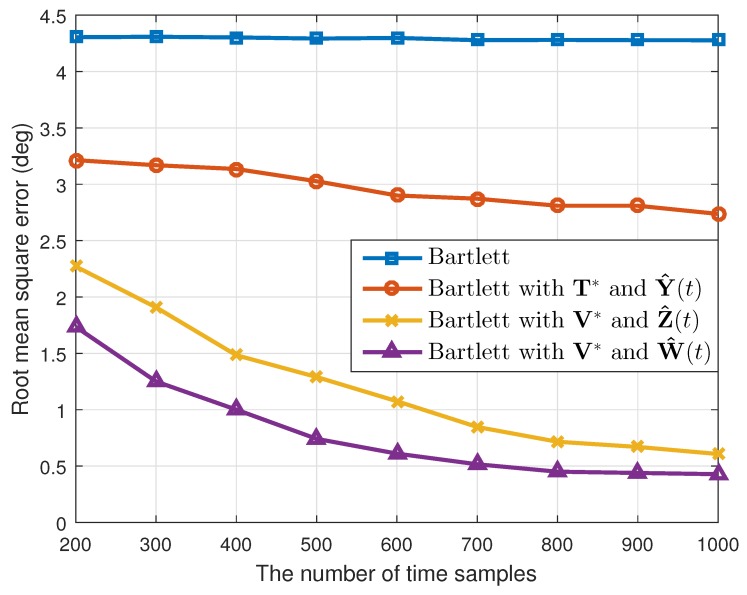
Root mean squared errors versus the number of time samples (SNR is 10 dB).

For the statistical performance evaluation, we calculated the resolution probability $P_{r}$ for the conventional Bartlett algorithm and the Bartlett with array interpolation methods. This probability is defined as:

(25) $$\begin{array}{c} P_{r}=\frac{N_{r}}{N_{t}}\times 100\;\left(\%\right) \end{array}$$

where $N_{r}$ indicates the number of times that two distinct DOAs were extracted from the received signals and $N_{t}$ denotes the number of simulations. Since we conducted this simulation 1000 times under the same conditions, $N_{t}$ became 1000. In addition, we calculated the root mean squared error (RMSE) defined as:

(26) $$\begin{array}{c} \mathrm{RMSE}=\sqrt{\frac{\sum_{k=1}^{K}\sum_{q=1}^{N_{t}}\left\{{\left(\theta_{k}-{\hat{\theta }}_{k}^{\left(q\right)}\right)}^{2}\right\}}{N_{t}}}\;{(}^{\circ }) \end{array}$$

where ${\hat{\theta }}_{k}^{\left(q\right)}$ is the estimated value of $\theta_{k}\;\left(k=1,\;2\right)$ in the $q_{th}\;\left(q=1,\;2\;\cdots ,\;N_{t}\right)$ simulation. When the number of the estimated targets was one, we used this as ${\hat{\theta }}_{k}^{\left(q\right)}$. The results are shown in Table 1. Considering the resolution probability and the RMSE, our proposed method performed better than the conventional Bartlett and the Bartlett with the transformation matrix $T^{*}$. In addition, while maintaining the simulation conditions, except the array SNR values, we calculated the resolution probability and the RMSE. As Figure 3 and Figure 4 show, our proposed method yielded good estimation results despite the different array SNR values. Moreover, after changing the number of time samples used to build the correlation matrix, a performance comparison among the interpolation methods was conducted, and the results of which are given in Figure 5 and Figure 6. Even though only a few time samples were used, our proposed array transformation showed improved estimation performance.

**Figure 7:**
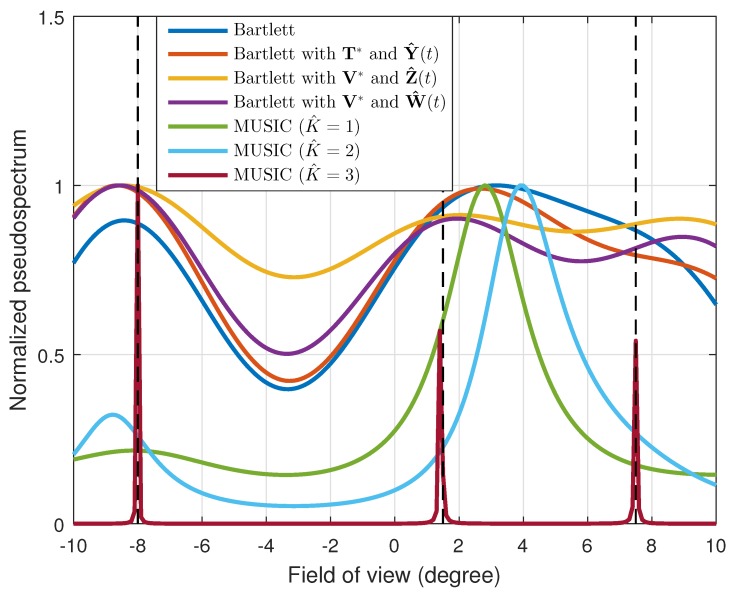
Normalized pseudospectrums for three targets located at $\left[-8^{\circ },\;1.5^{\circ },\;7.5^{\circ }\right]$.

We also conducted a simulation for a case in which three targets existed in the FOV of the radar. The simulation was conducted while maintaining the same simulation conditions given in Figure 1, except for the target information, and the result is shown in Figure 7. Here, the targets were located at $\left[\theta_{1},\;\theta_{2},\;\theta_{3}\right]=\left[-8^{\circ },\;1.5^{\circ },\;7.5^{\circ }\right]$. The conventional Bartlett and the Bartlett with the transformation matrix $T^{*}$ each estimated only two DOAs: $\left[-8.4^{\circ },\;3.2^{\circ }\right]$ and $\left[-8.6^{\circ },\;2.7^{\circ }\right]$, respectively. Thus, these methods failed to resolve the targets placed at $\left[\theta_{2},\;\theta_{3}\right]=\left[1.5^{\circ },\;7.5^{\circ }\right]$. However, when applying our proposed transformation matrix, we could identify the three different DOAs. Moreover, from the power calibrated interpolated received signal vector $\hat{W}\left(t\right)$, the DOAs were estimated as $\left[-8.6^{\circ },\;1.8^{\circ },\;8.8^{\circ }\right]$, which were the most exact estimated values. We also compared the performance of the proposed method to that of the multiple signal classification (MUSIC) algorithm, which is known as a high-resolution DOA estimation algorithm [19]. To apply the MUSIC algorithm, the number of targets must be estimated in advance using the Akaike information criterion or minimum description length [20,21]. If the number of targets is well estimated ($\hat{K}=3$), the most exact performance occurs. However, if the number is not accurately estimated (e.g., $\hat{K}=1$ or $\hat{K}=2$), the estimation performance deteriorates considerably, and it cannot be used as shown in Figure 7. In addition, since the MUSIC algorithm performs the eigenvalue decomposition and the multiplication of matrices spanned by the noise eigenvectors, additional computational complexity $O\left(N^{3}+N^{2}\times \left(2N-2L-1\right)\right)$ occurs compared to the conventional beamforming algorithm (i.e., the Bartlett method) [22,23]. Moreover, the Bartlett method is more robust to noise variance than the MUSIC algorithm [14]. Thus, for automotive radars, the Bartlett algorithm may be more appropriate for stably estimating the DOA of a target under noisy road environments.

Under the same simulation conditions, we also applied the total least squares estimation of signal parameters via rotational invariance techniques (TLS EPSRIT) [24]. The TLS ESPRIT method is one of subspace-based DOA estimation algorithms like the MUSIC and is a nonparametric DOA estimation method. When we used the TLS ESPRIT algorithm, the DOAs were estimated as $\left[-26.5^{\circ },\;-13.8^{\circ },\;12.7^{\circ }\right]$, which shows a large difference from the actual values. Because the TLS ESPRIT performs the eigenvalue decomposition three times to estimate the DOA, it requires more computation than our method. In addition, when using a small number of antenna elements, proper DOA estimation performance is not guaranteed with the ESPRIT method. Moreover, we compared the performance of the beamspace MUSIC algorithm [25] with that of our proposed method. We needed to find the approximate DOA of the target in the beamspace MUSIC method, which was an unnecessary process in our proposed method. After finding the approximate angle, a beamforming matrix was generated based on that angle. Generating the appropriate beamforming matrix is the most important point of the beamspace DOA estimation algorithms. For example, if the beamforming matrix was generated in the range of $-1^{\circ }$–$3^{\circ }$, the DOA was estimated as $1.8^{\circ }$, which was close to the real value. However, if the beamforming matrix was formed between $1^{\circ }$ and $5^{\circ }$, the DOA was estimated as $2.3^{\circ }$. In addition, when using beamspace DOA estimation algorithm, the algorithm had to be repeated as many times as the number of targets.

**Figure 8:**
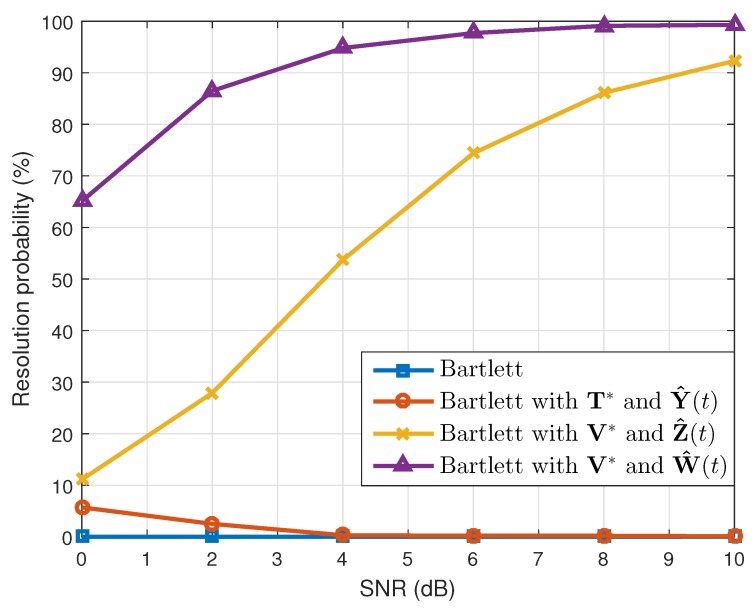
Resolution probabilities versus SNR (N=3).

**Figure 9:**
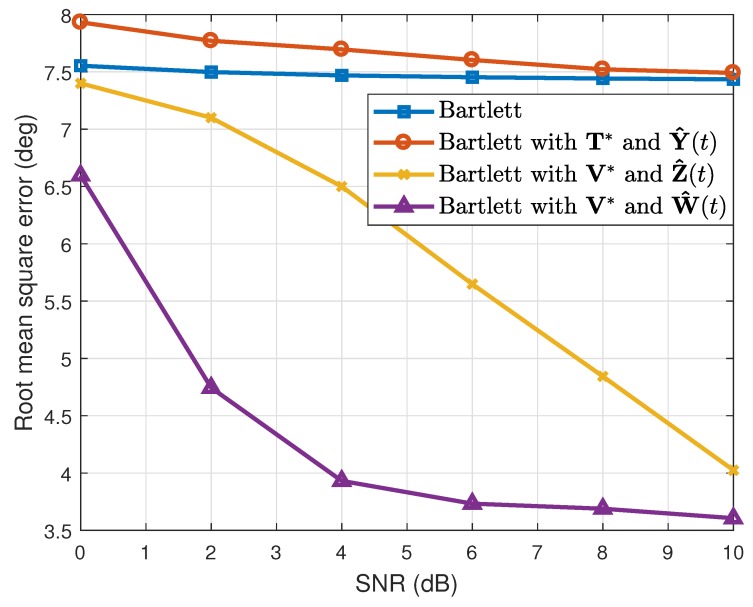
Root mean squared errors versus SNR (N=3).

**Figure 10:**
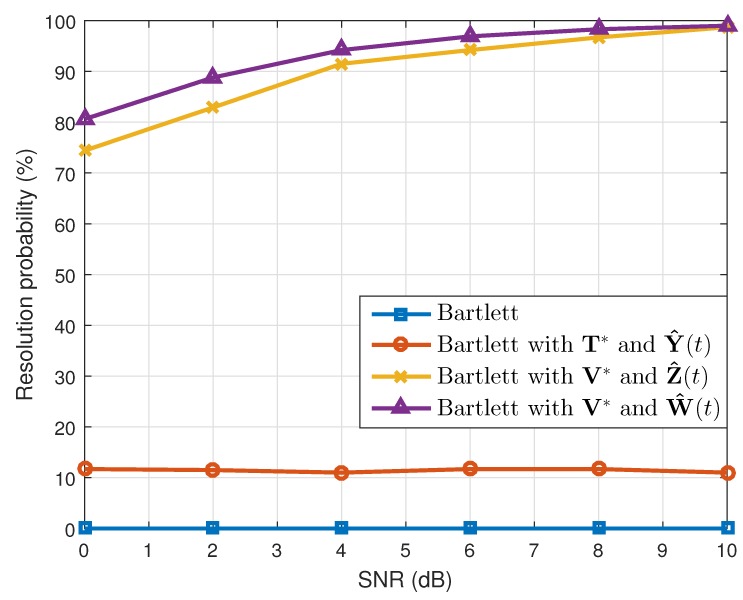
Resolution probabilities versus SNR (N=5).

**Figure 11:**
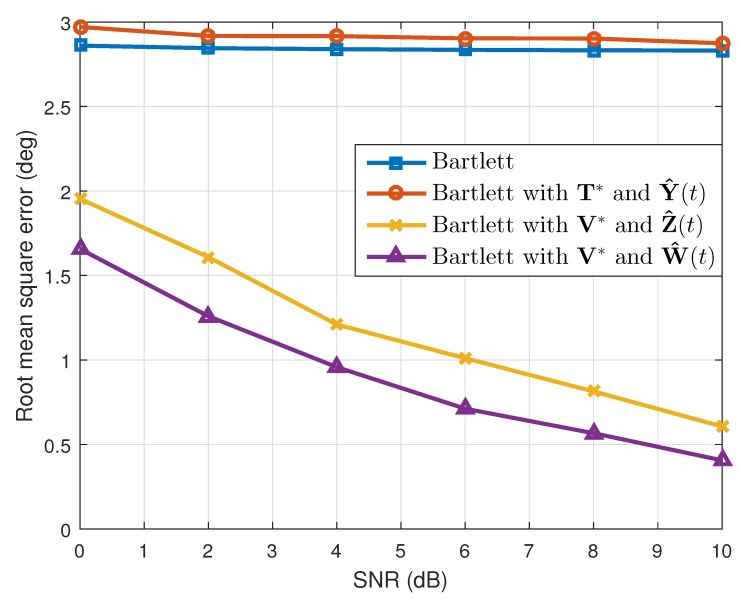
Root mean squared errors versus SNR (N=5).

Furthermore, simulations were conducted not only for the four array elements, but also for three and five array elements. When the number of array elements was three, the original location of the array elements was given as $\left[d_{1},\;d_{2},\;d_{3}\right]=\left[0,\;1.5\lambda ,\;3\lambda \right]$. This array was transformed to the minimum-redundancy array, and interpolated array elements were located at $\left[g_{1},\;g_{2},\;g_{3}\right]=\left[0,\;1\lambda ,\;3\lambda \right]$ [15,16]. In addition, we assumed that targets were located at $\left[\theta_{1},\;\theta_{2}\right]=\left[-4^{\circ },\;6.5^{\circ }\right]$ and that the FOV ranged from $-15^{\circ }$–$15^{\circ }$. Since the half-power beamwidth for the given array was $12^{\circ }$, the array had a very low angular resolution, and the given DOAs were difficult to distinguish from the conventional Bartlett algorithm. In addition, for the five array elements, the location of the original array elements was given as $\left[d_{1},\;d_{2},\;d_{3},\;d_{4},\;d_{5}\right]=\left[0,\;2.25\lambda ,\;4.5\lambda ,\;6.75\lambda ,\;9\lambda \right]$, and it was transformed to the location $\left[g_{1},\;g_{2},\;g_{3},\;g_{4},\;g_{5}\right]=\left[0,\;1\lambda ,\;4\lambda ,\;7\lambda ,\;9\lambda \right]$ [15,16]. For this case, the FOV was equal to that of the LRR, and targets were placed at $\left[\theta_{1},\;\theta_{2}\right]=\left[-1^{\circ },\;3^{\circ }\right]$. These DOAs were hard to separate out using the conventional Bartlett because the half-power beamwidth for the given array was $4.5^{\circ }$. For both cases of three and five array elements, the resolution probability and the RMSE were as given in Figure 8, Figure 9, Figure 10 and Figure 11, respectively, by increasing the array SNR from 0 dB–10 dB. As shown in the figures, our method also performed better for cases in which the number of array elements was three and five.

## 5. Measurement Results

To verify the performance of our proposed method, we also conducted actual measurements on a testing ground of the Mando Corporation using its automotive LRR. In the measurement, a single-element transmit antenna and four-element receiving uniform linear array antenna (N=4) were used, and the spacing between adjacent elements was 1.8λ. In addition, the half-power beamwidth of the array antenna was $7^{\circ }$, and the FOV of the LRR ranged from $-10^{\circ }$–$10^{\circ }$. This antenna system was equipped with an automotive radar and transmitted a 76.5-GHz frequency-modulated continuous wave signal. The transmitted signal was reflected from the front targets, and then, the reflected signals were received by the array antenna.

**Figure 12:**
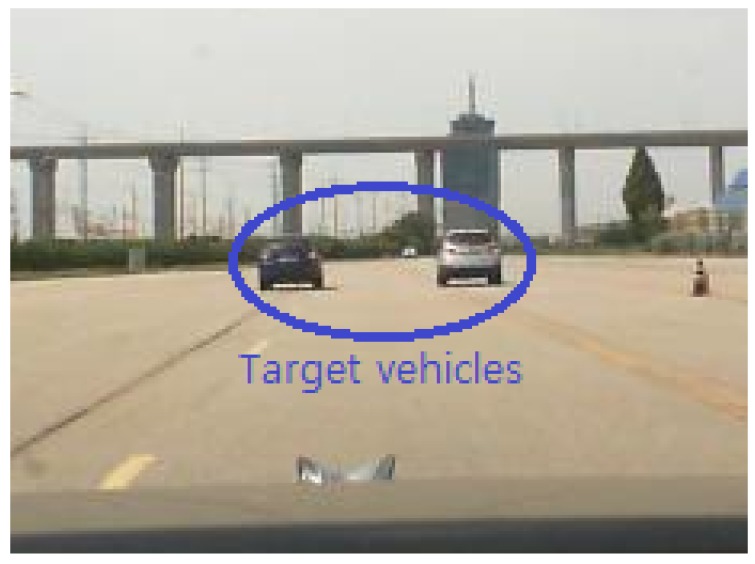
Measurement environment of two target vehicles located at $[-1.7^{\circ },\;4.6^{\circ }$].

Figure 12 shows the measurement environment. Two identical target vehicles were located at $\left[\theta_{1},\;\theta_{2}\right]=\left[-1.7^{\circ },\;4.6^{\circ }\right]$ and were 40 m away from a radar-equipped vehicle. In this measurement, we also used the Bartlett algorithm for the DOA estimation method and calculated the resolution probability and the RMSE for the original received signals and the interpolated received signals derived from the array interpolation methods. Under the same measurement environment, we recorded 600 radar scans. Thus, $N_{t}$ in (25) and (26) became 600 in this case. The results are listed in Table 2. Similar to the simulation results, based on both measures, the DOA estimation with the proposed transformation matrix $V^{*}$ showed better angular resolution and estimation accuracy than that of the conventional Bartlett and Bartlett method with the transformation matrix $T^{*}$. Furthermore, the estimation with $\hat{W}\left(t\right)$ showed the most improved resolution and estimation performance.

**Figure 13:**
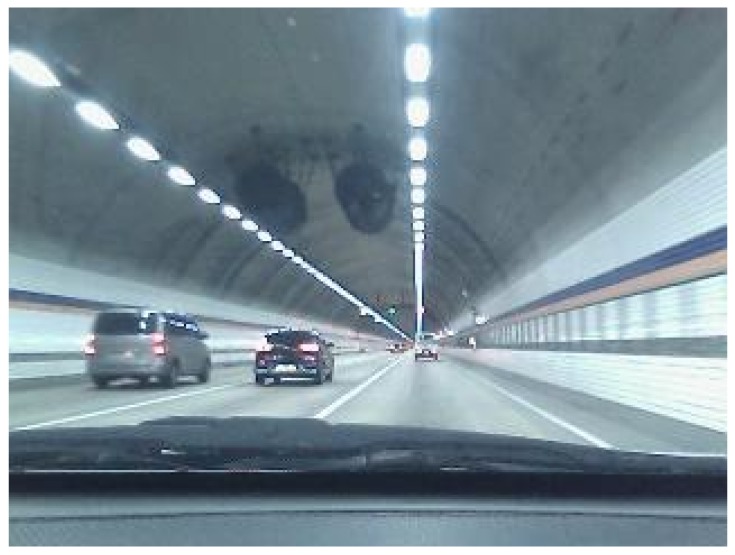
Measurement environment of two target vehicles (expressway).

Using the same automotive radar, the measurements were conducted on the expressway, as shown in Figure 13. In the experimental data, 100 radar scans were extracted when two targets were almost at the same distance and were close each other. For those cases, the DOA estimation methods were applied, and the results are listed in Table 3. Although the performance of the proposed algorithm was slightly lower than in the environment of Figure 12, the proposed algorithm showed improved angular resolution and lower RMSE over the other algorithms. It can be seen that the overall angular estimation performance degraded from the actual experimental measurement results rather than the simulation results. This was because the quality of the received signal was degraded due to the clutter caused by the surrounding road structures in an actual road environment [26,27]. In the environment shown in Figure 13, because the radar signals reflected from the wall of the tunnel were received with those from the desired targets, the quality of the received signal was inevitably deteriorated.

## 6. Conclusions

In this paper, we proposed a logarithmic-domain transformation matrix used for array interpolation to improve the accuracy of DOA estimation. Our transformation matrix was obtained by minimizing the differences between the phases of the original array elements and the interpolated array elements. Our proposed method identified a more accurate transformation matrix with less phase distortion, and the amplitudes of the array elements were maintained after the transformation. In addition, to improve the accuracy of the DOA estimation algorithm, we proposed a method for adjusting the powers of the interpolated received signals to a similar level. Finally, from the simulation and the measurement results, we verified that our new method showed much better angular resolution and estimation accuracy than did the DOA estimation using the conventional transformation matrix derived from the LLS method. The proposed method can be effectively applied to radar systems using a small number of antenna elements, such as automotive radar systems.

## Figures and Tables

**Table 1:** Resolution probabilities and root mean squared errors for two adjacent targets located at $\left[-3.5^{\circ },\;2.5^{\circ }\right]$.

DOA Estimation Method	$P_{r}$ (%)	RMSE (${}^{\circ }$)
Conventional Bartlett	0	4.28
Bartlett with $T^{*}$ and $\hat{Y}\left(t\right)$	74.9	2.67
Bartlett with $V^{*}$ and $\hat{Z}\left(t\right)$	99.4	0.64
Bartlett with $V^{*}$ and $\hat{W}\left(t\right)$	99.9	0.45

**Table 2:** Resolution probabilities and root mean squared errors for two target vehicles located at $\left[-1.7^{\circ },\;4.6^{\circ }\right]$.

DOA Estimation Method	$P_{r}$ (%)	RMSE (${}^{\circ }$)
Conventional Bartlett	0	5.72
Bartlett with $T^{*}$ and $\hat{Y}\left(t\right)$	0.67	6.20
Bartlett with $V^{*}$ and $\hat{Z}\left(t\right)$	46.7	4.65
Bartlett with $V^{*}$ and $\hat{W}\left(t\right)$	68.0	3.90

**Table 3:** Resolution probabilities and root mean squared errors for two target vehicles (expressway).

DOA Estimation Method	$P_{r}$ (%)	RMSE (${}^{\circ }$)
Conventional Bartlett	0	7.83
Bartlett with $T^{*}$ and $\hat{Y}\left(t\right)$	1.25	6.57
Bartlett with $V^{*}$ and $\hat{Z}\left(t\right)$	43.3	4.98
Bartlett with $V^{*}$ and $\hat{W}\left(t\right)$	65.1	4.21

## Abbreviations

The following abbreviations are used in this manuscript:

DOA	Direction of arrival
LLS	Linear least squares
FOV	Field of view
LRR	Long-range radar
SNR	Signal-to-noise ratio
RMSE	Root mean squared error
MUSIC	Multiple signal classification
TLS ESPRIT	Total least squares estimation of signal parameters via rotational invariance techniques

## Appendix A

First, ${\hat{y}}_{m}\left(t\right)$ and ${\hat{y}}_{m^{\prime }}\left(t\right)$ in (11) can be expressed as:

(A1) $$\begin{array}{ccc} {\hat{y}}_{m}\left(t\right) & = & \sum_{n=1}^{N}\left\{{T^{*}}_{\left(m,\;n\right)}\times x_{n}\left(t\right)\right\},\\ {\hat{y}}_{m^{\prime }}\left(t\right) & = & \sum_{n=1}^{N}\left\{{T^{*}}_{\left(m^{\prime },\;n\right)}\times x_{n}\left(t\right)\right\}. \end{array}$$

If we assume that received signals of the original array elements have nearly equal power such as:

(A2) $$\begin{array}{c} {\left|x_{n}\left(t\right)\right|}^{2}≅\gamma \geq 0\;\left(n=1,\;2,\;\cdots ,\;N\right), \end{array}$$

then the powers of each signal are given as:

(A3) $$\begin{array}{ccc} {\left|{\hat{y}}_{m}\left(t\right)\right|}^{2} & = & {\left|\sum_{n=1}^{N}\left\{{T^{*}}_{\left(m,\;n\right)}\times x_{n}\left(t\right)\right\}\right|}^{2}\;\;\;\\ & = & \gamma \times \sum_{n=1}^{N}{\left|{T^{*}}_{\left(m,\;n\right)}\right|}^{2}+\;2\sum_{\begin{array}{c} n=1,\\ n^{\prime }=1,\\ n\neq n^{\prime } \end{array}}^{N}ℜ\left\{{T^{*}}_{\left(m,\;n\right)}\bar{{T^{*}}_{\left(m,\;n^{\prime }\right)}}x_{n}\left(t\right)\bar{x_{n^{\prime }}\left(t\right)}\right\},\\ {\left|{\hat{y}}_{m^{\prime }}\left(t\right)\right|}^{2} & = & {\left|\sum_{n=1}^{N}\left\{{T^{*}}_{\left(m^{\prime },\;n\right)}\times x_{n}\left(t\right)\right\}\right|}^{2}\;\;\;\;\\ & = & \gamma \times \sum_{n=1}^{N}{\left|{T^{*}}_{\left(m^{\prime },\;n\right)}\right|}^{2}+\;2\sum_{\begin{array}{c} n=1,\\ n^{\prime }=1,\\ n\neq n^{\prime } \end{array}}^{N}ℜ\left\{{T^{*}}_{\left(m^{\prime },\;n\right)}\bar{{T^{*}}_{\left(m^{\prime },\;n^{\prime }\right)}}x_{n}\left(t\right)\bar{x_{n^{\prime }}\left(t\right)}\right\} \end{array}$$

where ℜ{·} is the operator that takes the real part of a complex number. Since:

(A4) $$\begin{array}{c} \sum_{n=1}^{N}{\left|{T^{*}}_{\left(m,\;n\right)}\right|}^{2}\neq \sum_{n=1}^{N}{\left|{T^{*}}_{\left(m^{\prime },\;n\right)}\right|}^{2},\;\;\;\;\;\;\\ {T^{*}}_{\left(m,\;n\right)}\bar{{T^{*}}_{\left(m,\;n^{\prime }\right)}}\neq {T^{*}}_{\left(m^{\prime },\;n\right)}\bar{{T^{*}}_{\left(m^{\prime },\;n^{\prime }\right)}}\;\;\;\\ (\mathrm{for}\;n\neq n^{\prime },\;n^{\prime }\in \left\{1,\;2,\;\cdots ,\;N\right\},\;\;\\ \;\mathrm{for}\;m\neq m^{\prime },\;m^{\prime }\in \left\{1,\;2,\;\cdots ,\;M\right\}), \end{array}$$

the powers of the interpolated received signals from the transformation matrix $T^{*}$ are not equivalent in all array elements.

In addition, the interpolated received signals in (17) can be expressed as:

(A5) $$\begin{array}{ccc} {\hat{z}}_{m}\left(t\right) & = & \prod_{n=1}^{N}{\left\{x_{n}\left(t\right)\right\}}^{{V^{*}}_{\left(m,\;n\right)}},\\ {\hat{z}}_{m^{\prime }}\left(t\right) & = & \prod_{n=1}^{N}{\left\{x_{n}\left(t\right)\right\}}^{{V^{*}}_{\left(m^{\prime },\;n\right)}}. \end{array}$$

Therefore, the powers of each term are given as:

(A6) $$\begin{array}{ccc} {\left|{\hat{z}}_{m}\left(t\right)\right|}^{2} & = & {\left|\prod_{n=1}^{N}{\left\{x_{n}\left(t\right)\right\}}^{{V^{*}}_{\left(m,\;n\right)}}\right|}^{2}\\ & = & {\left|{\left\{x_{1}\left(t\right)\right\}}^{{V^{*}}_{\left(m,\;1\right)}}\right|}^{2}\times \cdots \times {\left|{\left\{x_{N}\left(t\right)\right\}}^{{V^{*}}_{\left(m,\;N\right)}}\right|}^{2}\\ & = & \prod_{n=1}^{N}{\left|{\left\{x_{n}\left(t\right)\right\}}^{{V^{*}}_{\left(m,\;n\right)}}\right|}^{2},\\ {\left|{\hat{z}}_{m^{\prime }}\left(t\right)\right|}^{2} & = & {\left|\prod_{n=1}^{N}{\left\{x_{n}\left(t\right)\right\}}^{{V^{*}}_{\left(m^{\prime },\;n\right)}}\right|}^{2}\\ & = & {\left|{\left\{x_{1}\left(t\right)\right\}}^{{V^{*}}_{\left(m^{\prime },\;1\right)}}\right|}^{2}\times \cdots \times {\left|{\left\{x_{N}\left(t\right)\right\}}^{{V^{*}}_{\left(m^{\prime },\;N\right)}}\right|}^{2}\\ & = & \prod_{n=1}^{N}{\left|{\left\{x_{n}\left(t\right)\right\}}^{{V^{*}}_{\left(m^{\prime },\;n\right)}}\right|}^{2}. \end{array}$$

Since $\forall \;{V^{*}}_{\left(m,\;n\right)}\in R$ and $\forall \;{V^{*}}_{\left(m^{\prime },\;n\right)}\in R$, the above equation is redefined as:

(A7) $$\begin{array}{ccc} {\left|{\hat{z}}_{m}\left(t\right)\right|}^{2} & = & \prod_{n=1}^{N}{\left\{{\left|x_{n}\left(t\right)\right|}^{2}\right\}}^{{V^{*}}_{\left(m,\;n\right)}}\\ & = & \prod_{n=1}^{N}\gamma^{{V^{*}}_{\left(m,\;n\right)}}=\gamma^{\sum_{n=1}^{N}{V^{*}}_{\left(m,\;n\right)}},\\ {\left|{\hat{z}}_{m^{\prime }}\left(t\right)\right|}^{2} & = & \prod_{n=1}^{N}{\left\{{\left|x_{n}\left(t\right)\right|}^{2}\right\}}^{{V^{*}}_{\left(m^{\prime },\;n\right)}}\\ & = & \prod_{n=1}^{N}\gamma^{{V^{*}}_{\left(m^{\prime },\;n\right)}}=\gamma^{\sum_{n=1}^{N}{V^{*}}_{\left(m^{\prime },\;n\right)}}. \end{array}$$

However, because:

(A8) $$\begin{array}{c} \sum_{n=1}^{N}{V^{*}}_{\left(m,\;n\right)}\neq \sum_{n=1}^{N}{V^{*}}_{\left(m^{\prime },\;n\right)}\;\;\;\;\;\\ (\mathrm{for}\;m\neq m^{\prime },\;m^{\prime }\in \left\{1,\;2,\;\cdots ,\;M\right\}), \end{array}$$

the interpolated received signals have different powers for each element.
